# Genetic variations of cholesteryl ester transfer protein and diet interactions in relation to lipid profiles and coronary heart disease: a systematic review

**DOI:** 10.1186/s12986-017-0231-1

**Published:** 2017-12-08

**Authors:** Parvin Mirmiran, Zohre Esfandiar, Firoozeh Hosseini-Esfahani, Gelareh Koochakpoor, Maryam S. Daneshpour, Bahar Sedaghati-Khayat, Fereidoun Azizi

**Affiliations:** 1 Nutrition and Endocrine Research Center, Research Institute for Endocrine Sciences, Shahid Beheshti University of Medical Sciences, P.O.Box:19395-4763, Tehran, Iran; 2 Faculty of Nutrition Sciences and Food Technology, National Nutrition and Food Technology Research Institute, Shahid Beheshti University of Medical Sciences, Tehran, Iran; 3 Maragheh University of Medical Sciences, Maragheh, Iran; 4 Cellular Molecular and Endocrine Research Center, Research Institute for Endocrine Sciences, Shahid Beheshti University of Medical Sciences, Tehran, Iran; 5 Endocrine Research Center, Research Institute for Endocrine Sciences, Shahid Beheshti University of Medical Sciences, Tehran, Iran

**Keywords:** Cholesteryl ester transfer protein, Polymorphism, Lipids, Coronary heart disease, Diet, Nutrients

## Abstract

Data on diet–genotype interactions in the prevention or treatment of dyslipidemia have increased remarkably. This systematic review aimed to assess nutrigenetic studies regarding the modulating effect of diet on cholesteryl ester transfer protein (CETP) polymorphisms in relation to metabolic traits.

Data were collected through studies published between 2000 and SEP. 2016 using five electronic databases. The quality of eligible studies was assessed using a 12-item quality checklist, derived from the STrengthening the REporting of Genetic Association Studies (STREGA) statement. CETP variants that had associations with lipid profiles in previous studies were extracted for drawing of the linkage disequilibrium (LD) plot.

Among CETP variants, the rs9989419 best represented this genome wide association signal across all populations, based on LD r^2^ estimates from 1000 genomes references. In the 23 found eligible studies (clinical trials and observational), the TaqIB and I405V polymorphisms were the two most intensively studied. Two studies reported the effect of interaction between rs3764261 and diet on lipid levels. Regarding the rs708272 (Taq1B), individuals with the B1 risk allele showed better responses to dietary interventions than those with B2B2 genotype, whereas with I405V, inconsistent results have been reported. Modest alcohol consumption was associated with decreased risk of coronary heart disease among B2 carriers of rs708272.

It is concluded that variations in the CETP gene may modulate the effects of dietary components on metabolic traits. These results have been controversial, indicating complex polygenic factors in metabolic response to diet and lack of uniformity in the study conditions and designs.

**Electronic supplementary material:**

The online version of this article (10.1186/s12986-017-0231-1) contains supplementary material, which is available to authorized users.

## Background

Cardiovascular disease (CVD) is the leading cause of mortality worldwide [1]. Poor eating habits, smoking, and inactivity are environmental factors that increase the prevalence of CVD risk phenotypes, especially hypercholesterolemia [2–4]. Blood lipid concentrations play a role in the development of CVD. A meta-analysis concluds that levels of plasma low density lipoprotein cholesterol (LDL-C) and high density lipoprotein cholesterol (HDL-C) are autonomously correlated with CVD risk [5]. Evidence shows that the whole diet, particularly the quality and quantity of dietary fatty acids, affects plasma lipids. However, the extent of these dietary effects differs between individuals, usually owing to different eating habits or other inter-individual variations [6]. Gene–diet interactions may play an important role in the inter-individual diversity observed in plasma lipid concentrations and consequently on CVD risk [7–9]. Genetic susceptibility to dyslipidemia may modulate the association of dietary intake and metabolic syndrome [10].

A meta-analysis of genome-wide association studies have identified the association of cholesteryl ester transfer protein (CETP) genetic variants with blood lipid levels and CVD risk [11]; confirming strong associations of CETP rs3764261 and risk of HDL-C, LDL-C, total cholesterol (TC) and triglycerides (TG) in European, African and Asian populations; there are strong association of rs3764261, *P* < 1.0 × 10^−769^ with HDL-C concentration and effect size 0.241 per A allele [12]; this SNP has high linkage disequilibrium (LD) (r^2^ > 0.8) among multiple extracted CETP single nucleotide polymorphisms (SNPs) [12–15] and low LD with rs9989419 [16–18], rs1864163 [16] and rs1532624 [19]. In a meta-analysis, after adjustment of confounding factors, the Taq1B (rs708272) variant of CETP gene exhibited a significant association with HDL-C and coronary artery disease (CAD); the association between TaqIB genotype and CAD risk was also largely mediated through HDL-C plasma levels [20]; this confirmed SNP was reported to be in strong LD with the rs1800775 promoter polymorphism [11, 21] and in moderate LD with rs3764261 (r^2^ = 0.44) [22]; however this variant is not itself functional and it may represent a marker due to its LD with a functional variant [21, 23]. In a Chinese population optimum LD was reported for two groups of SNPs: rs3764261 and rs12149545; rs711752 and 708,272; also there was high LD for rs5882 and rs1801706 [24]. Strong significant associations were reported between CETP rs1532624 and HDL-C levels in Europeans and Mexicans, the *P*-values of which increased after adjustment of diet and physical activity in the model, indicating that this genetic effect may be mediated by environmental factors [14, 25]. Also some studies have reported that dietary habits might interact with these genetic variations in relation to dyslipidemia [6, 23, 26]. Regarding the magnitude of gene-diet interactions in the prevention or treatment of dyslipidemia in people with genetic risk factors and the increasing evidence linking CETP SNPs and dietary interactions, this systematic review aimed to document and discuss all studies investigating the effect of dietary modulation on the association of CETP gene and metabolic characteristics to summarize the scientific evidence available for individualized nutrition recommendations and to clarify how these interactions can be useful in updating public guidelines.

### Cholesteryl ester transfer protein (CETP)

**Fig. 1:**
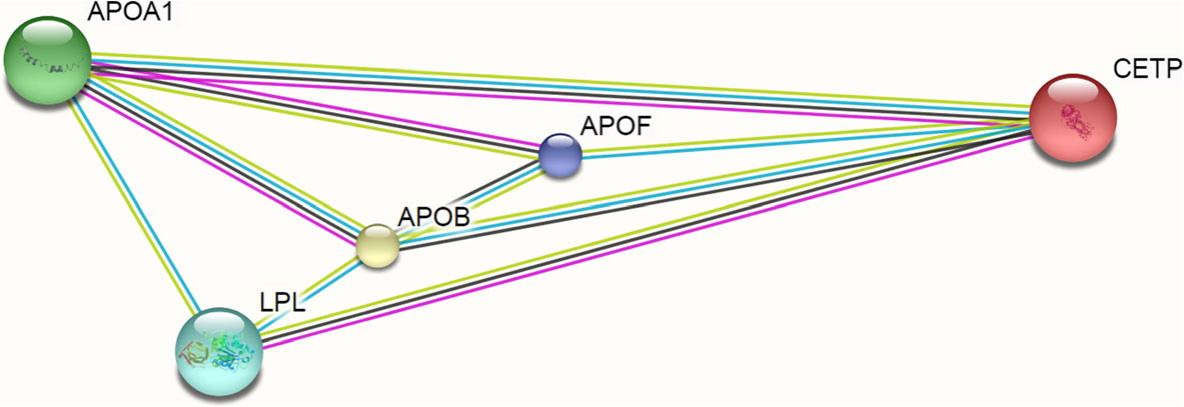
A network view of genetic associations. The cellular network showed LPL, APOB, APOA1 and APOF had the most gene interaction with CETP

The CETP gene is located in the q21 region of chromosome 16 and spans 25 kilobases genomic DNA encoding 16 exons. This gene yields a protein of 476 amino acids, forming a 74-kDa glycoprotein, the principal function of which is neutral lipid transfer between lipoproteins which eventually leads to lipoprotein remodeling such as modification of HDL size. CETP transfers cholesteryl esters from HDL to apolipoprotein B containing lipoproteins in exchange for triglycerides. CETP activity causes a decrease in plasma concentration of HDL-C and an increase in plasma concentration of LDL-C, which may increase the risk of CVD [27–29]. The cellular network demonstrated LPL, APOB, APOA1 and APOF had the most gene interactions (Fig. 1). The CETP gene is located in a highly polymorphic area in which several SNPs have been identified in coding and noncoding regions. Overall, this gene (including up-stream and down-stream) has more than 2800 SNPs, most of which do not have association reports.

## Methods

To obtain the information for markers, several databases were used including the Phenotype-Genotype Integrator (Phen-GenI), the NHGRI-EBI GWAS Catalog, ClinVar and GeneCards. In fact, these databases merge the information of several databases housed at the National Center for Biotechnology Information (NCBI), including Gene, dbGaP, OMIM, GTEx, dbSNP and the results of valid association reports in PubMed. A the end, all markers that had associations with lipid profiles were extracted for drawing of the LD plot (Additional file 1: Table S1).

**Fig. 2:**
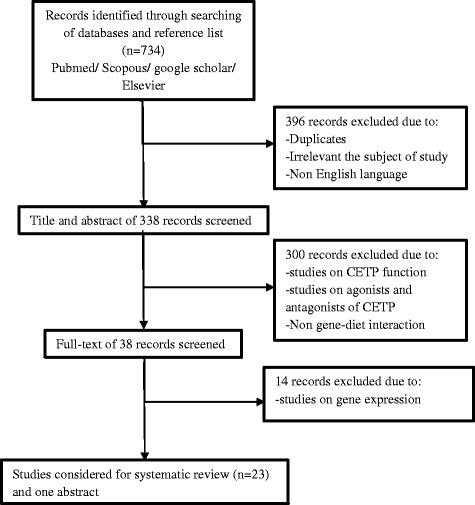
Flowchart of the study selection for inclusion in systematic review of genetic variations of Cholesteryl ester transfer protein (CETP) and diet interactions in relation to coronary heart disease and lipid profiles

For systematic reviewing of studies investigating the effect of CETP gene-diet interaction on lipid profiles or CVD, the search was limited to literature published between Jan. 2000 and Sep. 2016. Five electronic databases were searched, including PubMed, Google Scholar, ScienceDirect, Elsevier, and Scopus. The search was conducted using the following key words: (CETP OR cholesteryl ester transfer protein) AND (polymorphism* OR gene*) AND (diet* OR nutr* OR fat* OR protein* OR carbohydrate*). Eligibility criteria for a study to be included in this systematic review were investigations that evaluated the interaction between CETP polymorphism and dietary factors in relation to metabolic disease. The search included only articles published in the English language, with all types of study designs being included. We excluded papers focused on CETP function, agonists and antagonists of CETP, studies on gene expression, and studies on animal models; 23 studies were included with these parameters. Moreover the report of one article which was published in the abstract form was added to the tables [30]. Finally, 23 studies were included in this review based on data extraction and analysis of the quality of studies (Fig. 2).

To assess the quality of eligible studies, a 12-item quality checklist, derived from the STREGA statement (Strengthening the Reporting of Genetic Association Studies) [31], was used. A total quality score for each study was calculated by adding all the corresponding quality item scores (range: 0–12, higher scores indicating higher overall quality) (Table 1). All studies except three articles, explained their dietary assessment according to standard methods [32], including ≥2 days 24-h dietary recalls or dietary records before and after of interventional studies and food frequency questionnaire for observational studies.

**Table 1:** The Total Quality Score (TQS) of studies calculated bya 12 item quality checklist, derived from the STREGA statement

	Genotyping errors	Population stratificatin	Modelling haplotype variation	Hardy-Weinberg equilibrium	Replication	Selection of participants	Rationale for choice of genes	Treatment effects	Statistical methods	Relatednes of participant	Reporting of outcome data	Issues of data volume	TQS
Garcia-Rios A. et al., 2016 [47]	yes	no	no	yes	no	yes	yes	no	yes	no	yes	no	6
Mackay D.S. et al., 2015 [45]	yes	yes	yes	no	no	yes	yes	no	yes	no	no	no	6
Qi Q. et al., 2015 [22]	no	yes	no	yes	yes	yes	yes	yes	yes	no	yes	yes	8
Gammon C.S. et al., 2014 [23]	no	yes	yes	yes	yes	no	yes	yes	yes	no	yes	yes	9
Mehlig K. et al., 2014 [50]	no	no	no	no	yes	yes	yes	yes	yes	no	yes	yes	7
Rudkowska I. et al., 2013 [7]	yes	yes	yes	yes	no	yes	yes	no	yes	no	yes	no	8
Du J. et al., 2010 [34]	yes	yes	yes	yes	yes	yes	yes	yes	no	no	yes	yes	10
Corella D. et al., 2010 [35]	no	yes	yes	yes	yes	yes	yes	no	yes	no	yes	no	8
Corella D. et al., 2010 [48]	yes	no	yes	yes	yes	yes	yes	yes	yes	no	yes	yes	10
Darabi M. et al., 2009 [36]	no	yes	yes	yes	yes	yes	yes	yes	yes	no	yes	yes	10
Estévez-González M.D. et al., 2009 [33]	yes	yes	yes	yes	yes	no	yes	yes	no	no	yes	yes	9
Anagnostopoulou K.K. et al., 2009 [46]	no	yes	no	yes	yes	no	yes	no	yes	no	yes	no	6
Teran-Garcia M. et al.,2008 [37]	yes	no	no	no	yes	no	no	no	yes	yes	yes	yes	6
Jensen M.K. et al., 2008 [49]	yes	no	no	yes	yes	yes	yes	no	yes	no	yes	yes	8
Nettleton J.A. et al., 2007 [38]	no	yes	yes	yes	yes	yes	yes	yes	no	no	yes	yes	9
Li T.Y. et al., 2007 [42]	no	yes	yes	yes	yes	yes	yes	no	yes	no	yes	no	8
Tsujita Y. et al., 2007 [21]	yes	yes	yes	yes	yes	no	yes	yes	yes	no	yes	no	9
Aitken W.A.E. et al., 2006 [39]	yes	no	no	yes	yes	yes	yes	yes	no	no	yes	yes	8
Lottenberg A.M. et al., 2003 [43]	no	yes	yes	no	yes	yes	no	yes	yes	no	yes	yes	8
Plat J. et al., 2002 [40]	yes	no	yes	yes	yes	no	no	no	yes	no	no	no	5
Friedlander Y. et al., 2000 [6]	no	yes	yes	yes	yes	no	no	no	no	no	yes	yes	6
Wallace A.J. et al., 2000 [41]	yes	yes	yes	yes	yes	yes	yes	yes	no	no	yes	yes	10
Wallace A.J. et al., 2000 [26]	yes	yes	yes	yes	yes	yes	yes	yes	no	no	yes	yes	10

*STREGA* Strengthening the Reporting of Genetic Association Studies, *TQS* Total Quality Score

## Results

**Fig. 3:**
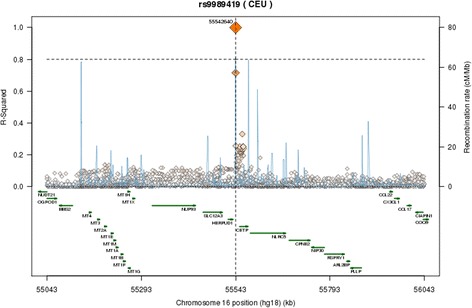
Linkage disequilibrium (LD) plot for single nucleotide polymorphisms (SNPs) encompassing the CETP region. Among CETP variants reported to be associated with lipid levels, the SNP rs9989419 had best represented this genome wide association signal across all populations based on LD r2 estimates from 1000 genome references

Among CETP variants reported to be associated with lipid levels, the SNP rs9989419 best represented this genome wide association signal across all populations based on LD r^2^ estimates from 1000 genomes references (Fig. 3).

Of the 23 full text articles and the one abstract found eligible, 16 studies were interventional (Table 2), and eight studies were observational (Tables 3-4); all of the studies assessed the genotype distribution for each CETP SNP and found them to be in Hardy-Weinberg equilibrium.

The subjects included in these studies consisted mostly of adult individuals (only one study included prepubertal children [33] but comprised different risk groups, including healthy [6, 7, 21, 26, 34–41], obese and overweight [22], hypercholesterolemic [33, 42–45], familial hypercholesterolemic [46], diabetic, and high CVD risk subjects [30, 47, 48]. Most studies included both sexes, but two studies assessed CETP gene and diet interaction only in male subjects [23, 42]. These studies were conducted in several different countries, including New Zealand [23, 26, 39, 41], Israel [6, 22], the United States [7, 38, 42, 49], Europe [30, 33, 35, 40, 46–48, 50], China [34, 51], Brazil [43, 44], Japan [21], Iran [36], and Canada [37, 45]. The studies examined the effects of the following individual or combinations of polymorphisms: Taq1B (17 studies), I405V (7 studies), rs3764261 (2 studies), C > T/In9 (one study), and rs183130 (1 study). In the context of genetic variation of CETP and dietary interactions, the TaqIB and then I405V polymorphisms were the two most intensively studied.

### Interventional studies

Sixteen intervention studies examined four of SNPs of the CETP gene: Taq1B (10 studies), I405V (6 studies), rs3764261 (2 studies), C > T/In9 (1 study). Eleven studies examined the effects of dietary fatty acids and the Mediterranean (Med) diet intervention on the relation of CETP variations and metabolic traits; other studies examined the effects of dietary interventions with kiwifruit and plant sterols on lipid profiles. The duration of the interventions varied from 6 days to 2 years (Table 2). Also of sixteen interventional studies, twelve studies found significant interaction of CETP polymorphisms (rs3764261, rs5882, rs708272, rs289714) and dietary factors in relation to plasma lipids.

**Table 2:** Selected intervention studies analyzing CETP gene variations and its interaction with diet in relation to plasma lipids and lipoproteins

Author	SNPs	Phenotypes evaluated	Dietary factor/ Method	Sample/Study duration	Interaction results		
					HDL-C	TG	Other lipid profiles
Qi Q. et al., 2015 [22]	rs3764261	HDL-C TG TC LDL-C	POUNDS LOST: high-fat (40%) and low-fat diet (20%), DIRECT: low-CHO (high-fat) and low-fat diet/ 5 day diet records and FFQ	POUNDS LOST trial, *n* = 732 DIRECT trial, *n* = 171 Overweight and obese subjects/ 2 years	CC genotype carriers, Increment of HDL-C (11.7 vs. 4.5%, P < 0.001) after intervention; CA/AA genotype carriers, no significant difference after intervention (Pi = 0.01)	CC genotype, more decrease in triglyceride levels after high-fat diet than the low-fat diet (−25.1 vs. -11.7%, *P* < 0.001), (Pi = 0.002)	TC: No interactionLDL-C: No interaction
Garcia-Rios A. et al., 2016 [47]	rs3764261	HDL-C TG	Med diet (35% fat, 22% MUFA) vs low fat diet (28% fat, 12% MUFA)/ questionnaire for adherence to the Med diet and FFQ	*N* = 424 MetS subjects/1 year	TT/TG genotype carriers, higher HDL-C levels compared to GG genotype after intervention (+2 vs 0 mg/dl, Pi = 0.006).	The TT/TG subjects, lower TG after intervention compared to GG participants (−31 mg/dl vs −16, Pi = 0.04). After low fat diet: no differences between genotypes	
Darabi M. et al., 2009 [36]	I405V rs5882	HDL-C LDL-C ApoA-1 Apo-B CETP	High-PUFA:SFA (1.2) and low-PUFA:SFA (0.3) diet	*N* = 85 normolipidemic students (62 men, 23 women)/cross-over: 2 dietary periods, 28 days	There was a trend for HDL-C (Pi = 0.06)	No interaction	APOA-I: VV/IV genotypes, greater reduction of ApoA-I after the low-P:S diet (−10, −11 vs −13 mg/dl, Pi = 0.016) than subjects with the II genotypesLDL-C and CETP: No interaction
Anagnostopoulou K.K. et al.,2009 [46]	I405V (rs5882)	HDL-C TG TC ApoA-1 Apo-B	Consuming fatty meal after 12 h fasting (oral fat tolerance test)/ -	41 men and 39 postmenopausal women heterozygous for familial hypercholestrolemia and 11 healthy control	Women carrying either the I or V allele compared with men carrying either the I or V allele had higher HDL-C (56 ± 20 vs. 38 ± 8 mg/dl, *P* < 0.01 and 53 ± 22 vs. 36 ± 7 mg/dl, *P* = 0.01), respectively	Women with the I allele had lower increment in TG-AUC compared to men carrying either the I or V allele (743 ± 363 vs. 934 ± 361 mg/dl, *P* = 0.04, 646 ± 285 vs. 844 ± 427 mg/dl, *P* = 0.05), respectively.	TC: Women with the I allele had lower TC levels compared to men carrying the I allele (315 ± 40 vs.305 ± 69 mg/dl, *P* = 0.04).ApoA-1: Women with the I allele had higher ApoA-I compared to men carrying either the I or V allele (158 ± 26 vs. 130 ± 23 mg/dl, *P* < 0.01, 169 ± 47 vs. 120 ± 23 mg/dl, P = 0.01), respectively.
Terán-García M. et al., 2008 [37]	I405V (rs5882)	HDL-C HDL- ApoA-1 HDL_2_ HDL_3_ TG, TC, LDL-C APO-B VLDL-C	Long term Overfeeding/Food record	*N* = 24 sedentary men (12 pairs of monozygotic twins) 100 days	Carriers of the IV/II genotypes compared to VV subjects, higher reduction in HDL-Apo AI (−7.0, −7.9 vs 2.7 mg/dl), HDL-C (−0.16, −0.12 vs 0.02 mmol/l), HDL_2_ (−0.04, −0.08 vs 0.03 mmol/l) and HDL_3_ (−0.13, −0.04 vs −0.004 mmol/l) levels	No interaction	TC, LDL-C, APO-B, VLDL-C: No interaction
Mackay D.S. et al., 2015 [45]	I405V rs5882	HDL-C TG, LDL-C	2 g/day plant sterol vs one meal a day with margarine/ -	*N* = 71 mildly hypercholesterolemic subjects (29 men and 42 women)/ Cross-over: 28 days	No interaction	After intervention, TG were lowered in homozygotes for the minor G-allele compared to A allele carriers (AA/AG) (−0.46 vs −0.03/−0.06 mmol/L, Pi = 0.014)	LDL-C: No interaction
Lottenberg A.M. et al., 2003 [43]	I405V (rs5882)	HDL-C TG TC LDL-C CETP	20 g/day margarine with or without 2.8 g/d dietary plant sterol ester/ Food record	*N* = 60 (50 women and 10 men) moderate primary hypercholesterolemia/Cross-over double blind: 2 dietary periods, 4 weeks	No interaction	No interaction	TC: There are significant percentage reduction (intervention group-placebo) in the II (−7.2) and in the IV (−4.2), but not in the VV genotype. LDL-C: There are only significant percentage reduction (control-placebo) in the II (−9.5) genotype.CETP: No interaction
Friedlander Y. et al., 2000 [6]	I405V (rs5882)	HDL-C TG TC LDL-C	High content of SFA and cholesterol (HSC) and low SFA and cholesterol (LSC) content diet/ Food record	*N* = 214 (108 males and 106 females)/ Cross-over: Two diet, 4 weeks	No interaction	No interaction	TC: No interactionLDL-C: No interaction
Gammon C.S. et al., 2014 [23]	Taq1B rs708272	HDL-C TG TG/HDL-C TC LDL-C ApoA-1, Apo-B	Healthy diet and Two green kiwi-fruit/day/ Food record and self reported diaries	*N* = 85 Hypercholesterolaemic men/Cross-over:8 weeks	TG/HDL-C ratio: B1/B1 homozygotes had lower TG:HDL-C (−0.23 (SD 0.58); P = 0·03) ratio after the intervention than the control group, whereas the ratio of B2 carriers was not affected (Pi = 0.03)	No interaction	No interaction
Du J. et al., 2010 [34]	Taq1B rs708272	HDL-C TG TC LDL-C ApoA-1, Apo-B-100	High carbohydrate, low fat diet (HC/LF) (15% fat and 70% carbohydrate) vs diets of 31% fat and 54% carbohydrate (washout diet)/ Daily dietary log	*N* = 56 Healthy young adults/Cross-over: 6 days Washout diet for 7 days	After washout diet, male carriers of B2 had higher HDL-C (54.0 ± 11.2 vs. 45.0 ± 7.3 mg/dl, *P* < 0.05) than males with B1B1. After HC/LF diet, male with B1B1 genotype had increased HDL-C (from 45.0 ± 7.3 to 49.8 ± 10.5 mg/dl, *P* < .05).	No interaction	LDL-C: After the HC/LF diet, a significant decreas in LDL-C both males (from 72.2 ± 25.0 to 55.2 ± 12.3 mg/dl, *P* < .05) and females (from 74.7 ± 14.2 to 65.0 ± 13.0 mg/dl, P < .05) with B1B1 was observedAPOA1: After washout diet, male carriers of B2 had higher APOA1 (176.3 ± 25.0 vs. 152.5 ± 24.4 mg/dl, P < 0.05) than males with B1B1. After HC/LF diet, male with B1B1 genotype had increased APOA1 (from 152.5 ± 24.4 to 158.1 ± 25.7 mg/dl, P < .05).
Dolores Estévez-González M. et al., 2009 [33]	Taq1B rs708272	HDL-C TG TC LDL-C ApoA-1, Apo-B	Two diets: skim milk and olive-oil-enriched skim milk / FFQ	*N* = 36 prepubertal children with mild hypercholesterolemi/Cross-over: 6 weeks	after the intake of olive-oil-enriched milk, HDL-C levels increased 0.090 mmol/l greater in the children with the B1B1 genotype than in those carrying at least 1 B2 allele (Pi = 0.049)	No interaction	TG, TC, LDL-C, APOA-1, APO B: No interaction
Anagnostopoulou K.K. et al.,2009 [46]	Taq1B rs708272	HDL-C TG, TC, ApoA-1, Apo-B	Consuming fatty meal after 12 h fasting (oral fat tolerance test)/ -	41 men and 39 postmenopausal women heterozygous for FH and 11 healthy control	No interaction	In the heterozygous FH-pathological subjects, the B2 allele carriers was related to lower levels of TG-AUC (Area under curve) (*P* = 0.01). In the heterozygous FH-normal subjects, the B1/B2 allele was not associated with TG-AUC levels (*P* = 0.55).	TC, ApoA-1, Apo-B: No interaction
Frances E. et al., 2006 [30] (Abstract)	Taq1B rs708272	Plasma lipids	Three diets, Med diet with olive oil; Med diet with nuts and control	*N* = 650 high risk subjects for cardiovascular disease/3 months	No interaction		
Aitken W.A.E. et al., 2006 [39]	Taq1B rs708272	HDL-C TG TC LDL-C	Two diets, high in SFA and high in PUFA/Food records and food recalls	35 individuals with the B1B1 genotype, age and sex-pair matched with B2 alleles carriers/Cross-over: 4 weeks	No interaction	No interaction	No interaction
Lottenberg A.M. et al., 2003 [43]	Taq1B (rs708272)	HDL-C TG TC LDL-C CETP	20 g/day margarine with or without 2.8 g/d dietary plant sterol ester/ Food record	*N* = 60 (50 women and 10 men) moderate primary hypercholesterolemia/Cross-over double blind: 2 dietary periods, 4 weeks	No interaction	No interaction	No interaction
Plat J.et al., 2002 [40]	Taq1B (rs708272)	HDL-C TG LDL-C	Vegetable-oil-derived plant stanols, wood-based plant stanols (3.8–4.0 g plant stanol esters a day) Control diet: rapeseed-oil-based margarine and shortening/FFQ	*N* = 112 (41 males and 71 females) healthy non-hypercholesterolaemic/ 8 weeks	No interaction	No interaction	LDL-C: There was a tendency to a greater decrement in LDL-C levels in the B1B1 subjects (−0·47 ± 0.35 mmol/L) compared to the B2B2 subjects (−0.31 ± 0.34 mmol/L); however, it was not significant (*P* = 0.123).
Wallace A.J. et al., 2000 [41]	Taq1B (rs708272)	HDL-C TG Dense LDL-C Light LDL-C	3 diets: standard lipid-lowering diet/ high SFA and high PUFA diets/ Diet records	*N* = 46 (23 men and 32 women)/Cross-over: 3/ 4 weeks	No interaction	No interaction	No interaction
Wallace A.J. et al., 2000 [26]	Taq1B (rs708272)	HDL-C TG TC LDL-C	3 diets: standard lipid-lowering diet/ high SFA and high PUFA diets/ Diet records	*N* = 55 (26–64 years)/Cross-over: 3 & 4 weeks	No interaction		TC: Individuals with the CETP B1B1 genotype showed an average 0.44 (95% CI: 0.22, 0.66) mmol:l greater change in total cholesterol than carriers of CETP B2 allele, comparing diets with high and low saturated fat.LDL-C: No interaction
Terán-García M. et al., 2008 [37]	C > T/In9 (rs289714)	HDL-C HDL-apo-A1 HDL-2 HDL-3 TG VLDL-C TC LDL-C LDL-apo-B	Long term Overfeeding/Food record	*N* = 24 sedentary men (12 pairs of monozygotic twins 100 days)	No interaction	No interaction	No interaction

*SNP* Single Nucleotide Polymorphism, *POUNDS LOST* Preventing Overweight Using Novel Dietary Strategies *DIRECT* Dietary Intervention Randomized Controlled Trial *MetS* Metabolic Syndrome, *TG* triglyceride, *TC* total cholesterol, *HDL-C* High density lipoprotein cholesterol, *LDL-C* Low density lipoprotein cholesterol, *PUFA:SFA* ratio of polyunsaturated to saturated fat *Med diet* Mediterranean Diet, *LCAT* lecithin-cholesterol acyltransferase, *CHO* Carbohydrate

Summarizing these findings, it appears that dietary intervention may modulate the effect of CETP gene variations on metabolic traits in different subjects. This effect was more significant when the influence of dietary fat on lipid concentration was investigated, although a consensus was not reached on this subject among the studies reviewed.

For the rs708272 (Taq1B) polymorphism, B1 homozygous genotype carriers had lower HDL-C concentrations than other genotypes, although individuals with the B1 allele showed a better response to the dietary intervention than those with the B2 allele; in other words, the dietary intervention was more effective in carriers of the B1 homozygous genotype. To be specific, dietary interventions aimed at improving blood lipid profiles are more effective for B1 homozygous carriers [23, 26, 30, 33, 34, 39–41, 43, 46]. For the rs5882 (I405V) polymorphism, studies reported inconsistent results, which may be because of the insufficient number of studies required to reach a comprehensive conclusion [6, 22, 37, 43, 45, 46].

### Observational studies

The effects of rs5882 and rs708272 CETP gene polymorphisms on dietary fatty acids, alcohol consumption, and adherence to Med diet were evaluated in eight observational studies; of them three studies found significant interaction of CETP gene polymorphisms and alcohol consumption in relation to coronary heart disease (CHD) (Table 3) and three others found significant interaction of CETP gene polymorphisms and alcohol or dietary fat intake in relation to total cholesterol (TC) and HDL-C (Table 4).

**Table 3:** Selected observational studies analyzing interaction of CETP gene variation with alcohol sonsumption in relation to CHD and lipid profiles

							Interaction results	
Author	SNPs	Phenotypes evaluated	Design	Sample	Dietary factor/Method	CHD	HDL & TG	Other lipid profiles
Mehlig K. et al., 2014 [50]	TaqIB (rs708272)	CHD	Case control	618 patients with CHD and 2921 controls	Alcohol consumption/ Self reported frequency of alcohol intake	Individuals with CETP TaqIB B2 homozygotes for intermediate ethanol intake had lower odds ratio than individuals with low ethanol intake (OR = 0.21; 95% CI: 0.10–0.44, Pi = 0.008).		
Corella D. et al., 2010 [35]	TaqIB (rs708272)	HDL-C TG TC LDL-C CHD	Nested case-control	*N* = 557 incident CHD cases and 1180 controls	Alcohol consumption/ Computerized diet history questionnaire	In drinkers, the B2B2 genotype associated with the greater risk of CHD (OR: 1.55, 95% CI: 1.05–2.29; *P* = 0.026), compared to non-drinkers. The greater CHD risk was reported in diabetic subjects carrying the B2 allele.	No interaction	No interaction
Jensen M.K. et al., 2008 [49]	TaqIB (rs708272)	HDL-C TG TC LDL-C CHD	Nested case-control	*N* = 505 incident CHD cases and 1010 controls	Alcohol consumption/ FFQ	The OR for CHD among individuals who drank 5–14.9 g/day (modest alcohol consumption) was 1.6 (95% CI: 1.1–2.3) for B1B1 and 0.7 (95% CI: 0.6–1.0) for B2 carriers (Pi = 0.02) in reference to non-drinkers	No interaction	No interaction

**Table 4:** Selected observational studies analyzing CETP gene variation and its interaction with diet in relation to lipid profiles

Author	SNPs	Phenotypes evaluated	Design	Sample	Dietary factor/Method	Main outcomes	
						HDL-C and TG	Other lipid profiles
Rudkowska I.et al., 2013 [7]	I405V (rs5882)	HDL-C TG TC LDL-C Apo-A1 Apo-B100	Cross-sectional	*N* = 553 (251 men and 322 women)	Dietary fat intake/ FFQ	No interaction	TC: TCconcentrations was higher in carriers of the TT genotype when consuming a high-total fat diet (TT: β = 0.0024 vs CT: β = −0.0029, CC: β = 0, Pi = 0.0460).LDL-C, Apo-A1, Apo-B100: No interaction
Corella D. et al., 2010 [48]	TaqIB (rs708272)	HDL-C	Cross-sectional	*N* = 4210 High CVD risk subjects	Alcohol, dietary fat, and MD diet/ Computerized diet history questionnaire	No interaction	
Nettleton J.A. et al., 2007 [38]	TaqIB (rs708272)	HDL-C TG LDL-C HDL_3_ Apo-B Apo-A	Cross-sectional	*N* = 11,559 (8764 Whites/2795 African Americans)	Dietary fat intake/ FFQ	No interaction	No interaction
Li T.Y. et al., 2007 [42]	TaqIB (rs708272)	HDL-C	Cohort study	*N* = 780 diabetic men (40–75 years)	Dietary fat intake/FFQ	HDL-C: There were an interaction between CETP TaqIB polymorphisms and total fat (high fat, B2B2: 44.9 vs B1B1: 36.2 mg/dl, *P* < 0.001, Pi = 0.003), animal fat (high animal fat, B2B2: 43.5 vs B1B1: 36.2 mg/dl, *P* < 0.001, Pi = 0.02), SFA (high SFA, B2B2: 43.8 vs B1B1: 36.2 mg/dl, P < 0.001, Pi = 0.02), and MUFA intakes (high MUFA, B2B2: 44.2 vs B1B1: 36.5 mg/dl, P < 0.001, Pi = 0.04) on HDL-C. No significant interaction was found between intakes of dietary cholesterol, vegetable fat and polyunsaturated fat and TaqIB polymorphism in determining HDL-C 1.51; B1B2: 1.58; B2B2: 1.63 mmol/L, *P* < 0.001) (Pi = 0.022).	
Corella D. et al., 2010 [48]	rs183130	HDL-C	Cross-sectional	*N* = 4210 High CVD risk subjects	Alcohol, dietary fat, and MD diet/ Computerized diet history questionnaire	HDL-C: No interaction	
Tsujita Y. et al., 2007 [21]	TaqIB (rs708272)	HDL-C TC	Cross-sectional	*N* = 1729	Alcohol consumption	In men carrying the B2B2 genotype and concuming alcohol, HDL-C concentrations were higher than those with the B1B1 genotype (*P* = 0.042). In woman with the B2B2 genotype, HDL-C concentrations were higher than other genotypes even after consuming alcohol (*P* < 0.001). In women with the B1B1 genotype and did not drink, HDL-C levels were lower than other genotypes (*P* < 0.001).	

*SNP* Single Nucleotide Polymorphism, *TG* triglyceride, *TC* total cholesterol, *CHD coronary heart disease*, *HDL-C* High Density Lipoprotein Cholesterol, *LDL-C* Low Density Lipoprotein Cholesterol, *MD* Mediterranean diet, *SFA* Saturated fatty acids, *MUFA* Mono-unsaturated fatty acids, *PUFA* Poly-unsaturated fatty acids, *FFQ* Food Frequency Questionnaire

Four studies examined the nutrigenetic effect of the Taq1B polymorphism and alcohol consumption in relation to HDL-C. In two studies by Corella et al., no significant interaction was found [35, 48], whereas Jensen et al., showed that the CETP Taq1B polymorphism modifies the relationship of alcohol intake with HDL-C concentrations [49]. Tsujita and colleagues performed a study on 1729 subjects, and found that male subjects who were homozygous for the B2 genotype and consumed ≥2 alcoholic drinks per day had higher HDL-C concentrations than those with the B1 homozygous genotype (*P* = 0.042), while no interaction was observed in men who consumed less than two drinks per day. Among women who did not drink, those who were homozygous for the B1 genotype had lower HDL-C concentrations than other genotypes (*P* < 0.001), while women who were homozygous for the B2 genotype and drank alcohol showed higher HDL-C concentrations than other genotypes [21]. Moderate alcohol consumption was associated with decreased risk of CHD in individuals homozygous for the B2 genotype of the Taq1B polymorphism [49, 50], while subjects with high alcohol intake, homozygous for the B2 genotype showed a greater risk of CHD compared with non-drinkers [35].

Four studies examined the relationship between CETP SNPs rs5882 and rs708272 and dietary fat. In studies by Nettleton et al. and Corella et al., no significant interaction was found [38, 48], whereas a cohort study of diabetic men found a strong association between CETP TaqIB and high intakes of monounsaturated fatty acids (MUFA), saturated fatty acids (SFA), animal fat, and total fat on plasma HDL-C levels (*P* values for the interaction effects = 0.04, 0.02, 0.02, and 0.003, respectively), an association which became stronger with increasing fat intake [42]. In a cross-sectional study that examined the effects of I405V polymorphisms, subjects with the IV genotype had higher TC than other genotypes when consuming higher fat intake [7].

## Discussion

The present systematic review of observational and clinical trial studies confirms that there are interactions between CETP polymorphisms and some foods, nutrients, or the Med diet in relation to plasma lipids and CHD.

To our knowledge this is the first review to address CETP gene-diet interactions in relation to lipid profiles and CHD.

Individuals homozygous for the B2 genotype who consumed alcohol, HDL-C concentrations were higher than those with the B1 homozygous genotype [21]. However, among alcohol drinkers, individuals homozygous for B2 had greater risk of CHD, even after adjusting for HDL-C concentrations. In addition, greater CHD risk was found in diabetic subjects carrying the B2 allele [35, 50]. However, one study has shown that anti-inflammatory capacity and HDL-particle size may be more important factors than high plasma HDL-C concentration because this concentration does not always protect against CHD [35]. The odds ratio for CHD among B2 allele carriers and modest alcohol consumption was lower than individuals with B1B1 genotypes. In a cohort study, the beneficial effects of the CETP TaqIB B2 allele on HDL-C concentrations were more evident in men with higher intakes of total fat, animal fat, SFAs, and MUFAs and in subjects with lower carbohydrate consumption [42].

Two studies reported the interaction between CETP rs3764261 and dietary fat or Med diet on lipid levels. These interactions may be due to their effect on CETP gene expression and changes in CETP mRNA. Moreover it has been found that high SFA diets increase CETP activity, compared to high MUFA and PUFA diets which may decrease this activity [22]. There were no significant differences among carriers of CETP polymorphisms after the consumption of a low-fat diet [47].

Significant differences were found in lipid profiles between CETP rs708272 genotype groups after dietary interventions, in which individuals homozygous for the B1 allele had lower concentrations of HDL-C and triglyceride (TG)/HDL-C ratio than carriers of B2 allele [23, 34], which was in line with the report of a pooled data analysis of 26 trials, indicating that the response of HDL-C to SFA was stronger in subjects with CETP Taq1B B2B2 genotypes than in those with other genotypes [52].

Some interventional studies did not observe these beneficial effects in regulating HDL-C concentrations or the interaction between CETP TaqIB genotype and changes in dietary fat in relation to HDL-C concentrations, although this may be a result of the small sample size resulting in limited power to detect statistical significance. The interactions between dietary fat and CETP polymorphisms in relation to HDL-C concentrations might be mediated through mutations modulating CETP activity [39].

There is only one published report examining the effect of the Taq1B polymorphism in children, which demonstrates that the polymorphism behaves similarly in children with mild hypercholesterolemia as in adults; individuals homozygous for the B1 genotype, who were found to have lower initial values of HDL-C, experienced a greater increase (0.09 mmol/l) in HDL-C levels after increasing their intake of MUFAs compared to the increase observed in B2 allele carrying subjects, this finding implies that a diet enriched with MUFAs can counteract the negative influence of the B1 homozygous genotype on HDL-C levels [33].

In an interventional study, subjects homozygous for the B1 genotype of the CETP Taq1B SNP who consumed regular daily portions of green kiwifruit showed improvement in their TG:HDL-C ratio. Considering that over 30% of the population had this genotype, this reduction could have improved CVD risk reduction guidelines [23].

Du et al. suggested that dietary components and ethnicity should be considered in studying the relationship of CETP TaqIB polymorphism with HDL-C in both genders; in their study, HDL-C and apoA-I increased after high carbohydrate/low fat dietary intervention only in males with the B1 homozygous genotype, which indicated that in this population of young, healthy Chinese subjects, males with the CETP TaqIB B1 homozygous genotype might be more susceptible to the effect of the high carbohydrate/low fat diet on HDL-C than males with the B2 allele and females with either genotype [34]. Using the oral fat tolerance test (OFTT), men carrying the B2 allele of the TaqIB polymorphism showed a higher postprandial TG peak and a delayed return to basal levels compared with women carrying the B2 allele. In contrast, in subjects with normal OFTT responses, no differences between the genders were found [46].

Three clinical trial studies found consistent results, i.e. the response in lipid profiles to plant sterol consumption may be influenced by a common genetic variant in CETP [40, 43, 45]. A potential genetic basis for the inter-individual variability in lipid profile responses to plant sterol consumption was found, with individuals having the G/G variant for rs5882 showing reductions in TG [45], total cholesterol, and LDL-C [43] concentrations. Interestingly, the same genotype was previously found to be associated with reduced CETP mass and activity [43, 53].

Furthermore, the CETP I405V polymorphism may affect responses of lipids and lipoproteins to changes in the dietary ratio of polyunsaturated to saturated fatty acids (P:S). Darabi et al. found that after low dietary P:S ratio intervention, unfavorable changes in apoA-I and HDL-C levels occurred in V allele carriers of the CETP I405V polymorphism, most likely due to effects of complex interactions between dietary fatty acids and CETP I405V on the serum lipid profile [36].

The CETP rs708272 polymorphism is located in the first intron of the CETP gene, indicating that it is very likely to have an adverse functional consequence on CETP activity. It is possible that this polymorphism is in LD with other mutations in the CETP promoter, which are known to have beneficial functional effects. It is possible that these promoter polymorphisms play a role in the mechanisms of the interaction [34] and the LD of CETP TaqIB polymorphism with the CETP-629CNA polymorphism might affect the expression of CETP. The mechanisms of the interaction between the CETP rs708272 polymorphism and dietary intake, especially fat consumption, in the regulation of lipid profiles, the expression level of CETP protein, and CETP protein activity have not been clarified [21, 23, 35, 42, 43].

Although many investigations have been conducted in this field, there is no consensus on this subject. The reasons for this are mainly polygenic principles in metabolic response to diet; many additional SNPs exist in genes influencing lipid metabolism that have not yet been examined. Additionally, there was significant diversity in study conditions and design among the reports, such as different sample sizes, duration, method of the dietary intervention, and characteristics of the population (i.e. healthy or illness-afflicted subjects, fasted or fed state of the participants), which is why we were unable to do a quantitative analysis (meta-analysis). More importantly, owing to the small sample size, patients were not divided based on genotype. All these factors make it difficult to reach comprehensive conclusions and obtain insights into disease-associated mechanisms.

Most of these studies conducted an analysis of isolated nutrients. However, analyses of dietary patterns provide a better context for studies that investigate the gene–diet interaction, which allows results to be generated with more confidence so that they can be generalized to larger populations. Additionally, there is a need to investigate other aspects that influence gene-diet interactions, such as physiological and environmental interactions (e.g. stress, smoking habits, physical activity, and sleep habits). More comprehensive conclusions can be obtained by analyzing each of these factors. Overall, the assessment of gene–diet interactions may be applicable for prediction of the impact of dietary changes on plasma lipid levels in order to make individualized nutrition recommendations to reduce the risk of CVD [54].

## Conclusion

Results of this review confirm that variations in the CETP gene may contribute to the effects of dietary components or dietary patterns on metabolic traits in different subjects.

## Additional file

Additional file 1: Table S1. Summary review of SNPs in the CETP gene with effects on plasma lipids. (DOCX 114 kb)

## Abbreviations

CAD: Coronary artery disease
CETP: Cholesteryl ester transfer protein
CHD: Coronary heart disease
CVD: Cardiovascular disease
HDL-C: High density lipoprotein cholesterol
LD: Linkage disequilibrium
LDL-C: Low density lipoprotein cholesterol
MUFA: Monounsaturated fatty acids
SFA: Saturated fatty acids
SNP: Single nucleotide polymorphism
STREGA: Strengthening the Reporting of Genetic Association Studies
TC: Total cholesterol
