# Supplementary material for: Genetic variations of cholesteryl ester transfer protein and diet interactions in relation to lipid profiles and coronary heart disease: a systematic review
Source: Nutr Metab (Lond). 2017 Dec 8;14:77. doi: 10.1186/s12986-017-0231-1 (PMC5721696; doi:10.1186/s12986-017-0231-1)
Supplement: Additional file 1: Table S1. — Summary review of SNPs in the CETP gene with effects on plasma lipids. (DOCX 114 kb) [file 12986_2017_231_MOESM1_ESM.docx]

**Additional File 1: Table S1. Summary review of SNPs in the CETP gene with effects on plasma lipids**

| **Author/Year** | **n** | **rs number** | **Allele** | **P-value**  **TC** | **P-value**  **HDL-C/Effect estimate** | | **P-value**  **TG/Effect estimate** | | **P-value**  **LDL-C/Effect estimate** |
| --- | --- | --- | --- | --- | --- | --- | --- | --- | --- |
| Heid IM/ 2008([1](#_ENREF_1)) | 1643, 4037, 9205 | rs1800775  rs9989419 | A/C  G/A |  | 6.05E-15 / -3.99 (mg/dl)  Pooled: 8.50E-27 | |  | |  |
| Kathiresan S/ 2008 ([2](#_ENREF_2)) | 2758, 5519, 7940, 5095 | rs1800775 |  |  | 1.0×10^-73^/ −0.18 (mg/dl) | |  | |  |
| Willer CJ, 2008 ([3](#_ENREF_3)) | 8656, 8072  8656, 3654  8656, 6981  8656, 7030  8656, 4881 | rs3764261  rs1864163  rs9989419  rs12596776  rs1566439 | A/C |  | 2.3×10^-57^/ 3.47 (mg/dl)  6.9×10^-39^ / 4.12  3.2×10^-31^/ 1.72  2.8×10^-8^/ 1.26  3.3×10^-8^/ 0.96 | |  | |  |
| Zemunik T, 2009 ([4](#_ENREF_4)) | 944 | rs7499892 | A |  | 9.0×10^−06^/ β=-0.235 | |  | |  |
| Ridker PM, 2009 ([5](#_ENREF_5)) | 18245 | rs1800775  rs1532624  rs708272  rs1864163  rs7499892  rs5880  rs1800777  rs12597002  rs4784744  rs5882  rs7202364  rs8051691  rs5883  rs4329913  rs1529929  rs2217332  rs13306677  rs247615  rs9989419  rs289744 | A/C  A/C  A/C  A/G  A/G  C/G  G/A  C/A  G/A  A/G  A/G  C/A  G/A  G/A  G/A  G/A  G/A  A/G  G/A  A/C |  | 3.7×10^-93^/ 3.09 (mg/dl per allele)  3.0×10^-92^/ 3.14  1.7×10^-90^/ 3.08  9.2×10^-71^/ -3.10  5.5×10^-70^/ -3.50  8.2×10^-25^/ -3.48  1.9×10^-21^/ -3.91  3.6×10^-17^/ -1.39  7.4×10^-17^/ -1.32  1.7×10^-15^/ 1.30  1.5×10^-10^/ 1.10  1.9×10^-10^/ 1.09  5.6×10^-9^/ 1.94  6.6×10^-9^/ -1.03  8.5×10^-9^/ 0.89  1.1×10^-8^/ -1.23  1.3×10^-8^/ 1.47  3.8×10^-8^/ -0.98  1.6×10^-45^/ -2.19  8.8×10^-17^/ 1.37 | |  | |  |
| Hiura Y, 2009([6](#_ENREF_6)) | 900, 3228 | rs3764261 | A/C |  | 1.9E–04/ β=5.6  3.4E–12/ β=6.2 | |  | |  |
|  |  | rs10945991 | A/C |  | 1.9E–07/ β=14.0  6.0E–03/ β=4.5 | |  | |  |
| Aulchenko YS, 2009 ([7](#_ENREF_7)) | 17797, 22562 | rs1532624 |  | 0.01, β=-0.028 | 9.4E-94/ β=-0.213 | | 1.1E-3, β=0.038 | | 3.3E-3, β=0.034 |
| **Table 1: Continued** | | | | | | | | | |
| **Author/Year** | **n** | **rs number** | **Allele** | **P-value**  **TC** | | **P-value**  **HDL-C/Effect estimate** | | **P-value**  **TG/Effect estimate** | **P-value**  **LDL-C/Effect estimate** |
| Zabaneh D, 2010 ([8](#_ENREF_8)) | 4560 | 3764261  9989419 | A/C  G/A |  | | 1.3×10^-48^/ OR=0.07  1.4×10^-20^/ OR=-0.05 | |  |  |
| Wu Y, 2013 ([9](#_ENREF_9)) | 1782, 1719 | rs1800775 | A/C |  | | 3.4E-09/ β=0.04 | |  |  |
| Smith EN, 2010 ([10](#_ENREF_10)) | 12,163 | rs247616 | T/C |  | | 9.7×10^-24^/ β=2.99 | |  |  |
| Waterworth DM, 2010 ([11](#_ENREF_11)) | 17723, 37774 | rs9989419  rs16965220 |  |  | | 1.3×10^-32^/ β=0.035  0.04/ β=-0.006 | | 0.67/ β=0.003  9.6×10^-6^/ β=0.028 | 0.58/ β=-0.002  0.01/ β=0.009 |
| Igl W, 2010 ([12](#_ENREF_12)) | 656, 3,282 | rs9866473 |  |  | | 2.55E-04 | |  |  |
|  |  | rs9863761 |  |  | | 7.74E-06 | |  |  |
|  |  | rs2124147 |  |  | | 2.23E-04 | |  |  |
|  |  | rs1532624 | A/C |  | | 2.55E-06/ 0.48  1.96E-09/ 0.57 (standardized) | |  |  |
| Kraja AT, 2011([13](#_ENREF_13)) | 22161 | 173539 | A>C |  | | HDLC/TG  4.5E-16 / β=0.26 | |  |  |
| Middelberg RPS, 2011 ([14](#_ENREF_14)) | 2548 adolescents and 9145 adults | rs173539 | T>C |  | | 1.7×10^-40^/ β=0.255 | |  |  |
| Weissglas-Volkov D, 2013 ([15](#_ENREF_15)) | 6073 | rs1532624 | A/C |  | | 1.39E-24/ OR=0.23 | |  |  |
| Coram MA, 2013 ([16](#_ENREF_16)) | 8153, 3587, 7138 | rs247617  rs247617 | A/C |  | | 1.48×10^-44^  3.48×10^-16^ | |  |  |
| Ko A, 2014 ([17](#_ENREF_17)) | 19273 | rs5880  rs118146573 |  | 3.79×10^-10^/ β=-0.20 | | 1.76×10^-16^/ β=-0.29 | |  |  |
| Zhou L, 2013 ([18](#_ENREF_18)) | 3451,  8830 | rs3764261 | T/G |  | | 6.65×10^-12^/ 0.086 mg/dl  2.0×10^-15^/ 0.062 mg/dl | |  |  |
| Kathiresan S, 2009 ([19](#_ENREF_19)) | 19840  20623 | rs173539 | C/T |  | | 4.0×10^-75^/ 0.25 mg/dl | |  |  |
| Sabatti C, 2009 ([20](#_ENREF_20)) | 4763 | rs3764261 | T/G |  | | 6.97E-29/ β= 0.092 | |  |  |
| Willer CJ, 2013 ([21](#_ENREF_21)) | 177533  83626 | rs3764261  rs3764261 | T/G | 4×10^-31^/0.050 | | 1.4×10^-769^ /0.241 | | 2×10^-25^/0.040 | 2×10^-34^/-0.053 |
| Lu X, 2016 ([22](#_ENREF_22)) | 8344, 14739 | rs3764261 |  | 9.69×10^–23^/ β= 4.439 | | 2.00×10^–97^/ β= 3.353 | |  |  |
| Below JE, 2016 ([23](#_ENREF_23)) | 4383, 7876 | rs7499892 | T/C |  | | 4.16E-17/ β= 0.233  3.80E-49/ β= 0.243 | |  |  |
| Kettunen J, 2017 ([24](#_ENREF_24)) | 8330 | rs3764261 | A/C |  | | 1.23×10^-36^/ β=0.22 | |  |  |
| Nagy R, 2017 ([25](#_ENREF_25)) | 20,032 | rs3764261 | A/C |  | | 1.40E-113 | |  |  |

References:

1. Heid IM, Boes E, Muller M, Kollerits B, Lamina C, Coassin S, et al. Genome-wide association analysis of high-density lipoprotein cholesterol in the population-based KORA study sheds new light on intergenic regions. Circulation Cardiovascular genetics. 2008;1(1):10-20.

2. Kathiresan S, Melander O, Guiducci C, Surti A, Burtt NP, Rieder MJ, et al. Six new loci associated with blood low-density lipoprotein cholesterol, high-density lipoprotein cholesterol or triglycerides in humans. Nature genetics. 2008;40(2):189-97.

3. Willer CJ, Sanna S, Jackson AU, Scuteri A, Bonnycastle LL, Clarke R, et al. Newly identified loci that influence lipid concentrations and risk of coronary artery disease. Nature genetics. 2008;40(2):161-9.

4. Zemunik T, Boban M, Lauc G, Janković S, Rotim K, Vatavuk Z, et al. Genome-wide Association Study of Biochemical Traits in Korčula Island, Croatia. Croatian Medical Journal. 2009;50(1):23-33.

5. Ridker PM, Pare G, Parker AN, Zee RY, Miletich JP, Chasman DI. Polymorphism in the CETP gene region, HDL cholesterol, and risk of future myocardial infarction: Genomewide analysis among 18 245 initially healthy women from the Women's Genome Health Study. Circulation Cardiovascular genetics. 2009;2(1):26-33.

6. Hiura Y, Shen CS, Kokubo Y, Okamura T, Morisaki T, Tomoike H, et al. Identification of genetic markers associated with high-density lipoprotein-cholesterol by genome-wide screening in a Japanese population: the Suita study. Circulation journal : official journal of the Japanese Circulation Society. 2009;73(6):1119-26.

7. Aulchenko YS, Ripatti S, Lindqvist I, Boomsma D, Heid IM, Pramstaller PP, et al. Loci influencing lipid levels and coronary heart disease risk in 16 European population cohorts. Nature genetics. 2009;41(1):47-55.

8. Zabaneh D, Balding DJ. A genome-wide association study of the metabolic syndrome in Indian Asian men. PloS one. 2010;5(8):e11961.

9. Wu Y, Marvelle AF, Li J, Croteau-Chonka DC, Feranil AB, Kuzawa CW, et al. Genetic association with lipids in Filipinos: waist circumference modifies an APOA5 effect on triglyceride levels. Journal of lipid research. 2013;54(11):3198-205.

10. Smith EN, Chen W, Kahonen M, Kettunen J, Lehtimaki T, Peltonen L, et al. Longitudinal genome-wide association of cardiovascular disease risk factors in the Bogalusa heart study. PLoS genetics. 2010;6(9):e1001094.

11. Waterworth DM, Ricketts SL, Song K, Chen L, Zhao JH, Ripatti S, et al. Genetic variants influencing circulating lipid levels and risk of coronary artery disease. Arteriosclerosis, thrombosis, and vascular biology. 2010;30(11):2264-76.

12. Igl W, Johansson A, Wilson JF, Wild SH, Polasek O, Hayward C, et al. Modeling of environmental effects in genome-wide association studies identifies SLC2A2 and HP as novel loci influencing serum cholesterol levels. PLoS genetics. 2010;6(1):e1000798.

13. Kraja AT, Vaidya D, Pankow JS, Goodarzi MO, Assimes TL, Kullo IJ, et al. A bivariate genome-wide approach to metabolic syndrome: STAMPEED consortium. Diabetes. 2011;60(4):1329-39.

14. Middelberg RP, Ferreira MA, Henders AK, Heath AC, Madden PA, Montgomery GW, et al. Genetic variants in LPL, OASL and TOMM40/APOE-C1-C2-C4 genes are associated with multiple cardiovascular-related traits. BMC medical genetics. 2011;12:123.

15. Weissglas-Volkov D, Aguilar-Salinas CA, Nikkola E, Deere KA, Cruz-Bautista I, Arellano-Campos O, et al. Genomic study in Mexicans identifies a new locus for triglycerides and refines European lipid loci. Journal of medical genetics. 2013;50(5):298-308.

16. Coram MA, Duan Q, Hoffmann TJ, Thornton T, Knowles JW, Johnson NA, et al. Genome-wide characterization of shared and distinct genetic components that influence blood lipid levels in ethnically diverse human populations. American journal of human genetics. 2013;92(6):904-16.

17. Ko A, Cantor RM, Weissglas-Volkov D, Nikkola E, Reddy PM, Sinsheimer JS, et al. Amerindian-specific regions under positive selection harbour new lipid variants in Latinos. Nature communications. 2014;5:3983.

18. Zhou L, He M, Mo Z, Wu C, Yang H, Yu D, et al. A genome wide association study identifies common variants associated with lipid levels in the Chinese population. PloS one. 2013;8(12):e82420.

19. Kathiresan S, Willer CJ, Peloso GM, Demissie S, Musunuru K, Schadt EE, et al. Common variants at 30 loci contribute to polygenic dyslipidemia. Nature genetics. 2009;41(1):56-65.

20. Sabatti C, Service SK, Hartikainen AL, Pouta A, Ripatti S, Brodsky J, et al. Genome-wide association analysis of metabolic traits in a birth cohort from a founder population. Nature genetics. 2009;41(1):35-46.

21. Willer CJ, Schmidt EM, Sengupta S, Peloso GM, Gustafsson S, Kanoni S, et al. Discovery and refinement of loci associated with lipid levels. Nature genetics. 2013;45(11):1274-83.

22. Lu X, Huang J, Mo Z, He J, Wang L, Yang X, et al. Genetic Susceptibility to Lipid Levels and Lipid Change Over Time and Risk of Incident Hyperlipidemia in Chinese Populations. Circulation Cardiovascular genetics. 2016;9(1):37-44.

23. Below JE, Parra EJ, Gamazon ER, Torres J, Krithika S, Candille S, et al. Meta-analysis of lipid-traits in Hispanics identifies novel loci, population-specific effects, and tissue-specific enrichment of eQTLs. Scientific reports. 2016;6:19429.

24. Kettunen J, Demirkan A, Wurtz P, Draisma HH, Haller T, Rawal R, et al. Genome-wide study for circulating metabolites identifies 62 loci and reveals novel systemic effects of LPA. Nature communications. 2016;7:11122.

25. Nagy R, Boutin TS, Marten J, Huffman JE, Kerr SM, Campbell A, et al. Exploration of haplotype research consortium imputation for genome-wide association studies in 20,032 Generation Scotland participants. Genome medicine. 2017;9(1):23.
